# Killer Cell Immunoglobulin-like Receptors and Their HLA Ligands are Related with the Immunopathology of Chagas Disease

**DOI:** 10.1371/journal.pntd.0003753

**Published:** 2015-05-15

**Authors:** Christiane Maria Ayo, Pâmela Guimarães Reis, Márcia Machado de Oliveira Dalalio, Jeane Eliete Laguila Visentainer, Camila de Freitas Oliveira, Silvana Marques de Araújo, Divina Seila de Oliveira Marques, Ana Maria Sell

**Affiliations:** 1 Post Graduation Program of Biosciences Applied to Pharmacy, Department of Analysis Clinical and Biomedicine, Maringa State University, Maringa, Parana, Brazil; 2 Basic Health Sciences, Maringa State University, Maringa, Parana, Brazil; 3 Medical Clinic Department, Londrina State University, Londrina, Parana, Brazil; Federal University of São Paulo, BRAZIL

## Abstract

The aim of this study was to investigate the influence of killer cell immunoglobulin-like receptor (KIR) genes and their human leucocyte antigen (HLA) ligands in the susceptibility of chronic Chagas disease. This case-control study enrolled 131 serologically-diagnosed Chagas disease patients (59 men and 72 women, mean age of 60.4 ± 9.8 years) treated at the University Hospital of Londrina and the Chagas Disease Laboratory of the State University of Maringa. A control group was formed of 165 healthy individuals - spouses of patients or blood donors from the Regional Blood Bank in Maringa (84 men and 81 women, with a mean age of 59.0 ± 11.4 years). Genotyping of HLA and KIR was performed by PCR-SSOP. KIR2DS2-C1 in the absence of KIR2DL2 (KIR2DS2^+^/2DL2^-^/C1^+^) was more frequent in Chagas patients (*P* = 0.020; *Pc* = 0.040; OR = 2.14) and, in particular, those who manifested chronic chagasic cardiopathy—CCC (*P* = 0.0002; *Pc* = 0.0004; OR = 6.64; 95% CI = 2.30–18.60) when compared to the control group, and when CCC group was compared to the patients without heart involvement (*P* = 0.010; *Pc* = 0.020; OR = 3.97). The combination pair KIR2DS2^+^/2DL2^-^/KIR2DL3^+^/C1^+^ was also positively associated with chronic chagasic cardiopathy. KIR2DL2 and KIR2DS2 were related to immunopathogenesis in Chagas disease. The combination of KIR2DS2 activating receptor with C1 ligand, in the absence of KIR2DL2, may be related to a risk factor in the chronic Chagas disease and chronic chagasic cardiopathy.

## Author Summary

Chagas disease is an infection caused by the haemoflagellate protozoan *Trypanosoma cruzi*. It is one of the most important public health problems in Latin America, and was first described by Carlos Justiniano Ribeiro das Chagas, a Brazilian physician and scientist, in 1909. It is mostly vector-borne transmitted to humans by contact with faeces of triatomine bugs. The World Health Organization estimates that about 6 to 7 million people are currently infected with *T*. *cruzi* worldwide. The disease is characterised by acute and chronic phases. The immune response during disease development is crucial for protection because immunological imbalances can lead to heart and digestive tract lesions in chagasic patients. In this work we analysed the role of receptors of immune cells known as Natural Killer cells (killer cell immunoglobulin-like receptor—KIR) and their ligands (Human leukocyte antigens—HLA) in chagasic patients compared to healthy individuals. The uncontrolled activation of NK cells can lead to tissue damage, which, in turn, leads to the development of serious chronic illness. We found that KIR-HLA complex may be related to a risk factor in the chronic Chagas disease and chronic chagasic cardiopathy.

## Introduction

Chagas disease, caused by the flagellate parasite *Trypanosoma cruzi*, currently affects around 6 million to 7 million people worldwide and about 25 million have the potential risk of becoming infected [1]. The disease is endemic and its development occurs in acute and chronic phases. The latter may present in different clinical forms, indeterminate, cardiac and/or digestive, with the chronic cardiac form being the most serious [2]. This variation in pathological manifestation has been reported to be related to the complexity of parasite and to differences in host immune response, such as the ability to control parasitaemia, the strength of inflammatory reactions, and the induction of autoimmune-like responses [3–7]. Tissue damage resulting from inflammatory infiltrates and persistence of *T*. *cruzi* in myocardial tissue and changes in microcirculation and commitment of the autonomic nervous system are involved in the pathogenesis of cardiomyopathy. However, the precise pathogenic mechanism of Chagas' heart disease is not completely elucidated [7, 8].

The inflammatory process in the chronic phase of Chagas disease shows signs of cellular activity with CD4^+^ T and CD8^+^ T lymphocytes in heart tissue; though fewer numbers of natural killer (NK) cells, macrophages and B cells are also present [9, 10]. In asymptomatic or indeterminate chronic Chagas disease, the presence of circulating NK cells (CD3^-^CD16^+^CD56^+^ and CD3^-^CD16^+^CD56^dim^) coupled with the presence of immunoregulatory (Treg^-^CD4^+^CD25^high^ and NKT^-^CD3^+^CD16^-^CD56^+^) or macrophage-like cells (CD14^+^CD16^+^) are responsible for the control the inflammatory mechanisms. However, failure in immunoregulatory mechanisms, with basal levels of NK, NKT and CD4^+^CD25^high^ cells, associated with an increased expression of activated CD8^+^ T cells, are associated with heart disease [11, 12, 13]. The effective function of NK cells is regulated by a balance of activating and inhibitory signals mediated by a diverse set of receptors expressed on their surface, including killer immunoglobulin-like receptors (KIR), which recognize and bind in HLA class I molecules present on the surface of the target cells [14].

KIR is a family of 15 closely linked genes and highly polymorphic, on chromosome 19q13.4, that encodes both inhibitory and activating receptors. The receptors molecules may have two or three immunoglobulin-like domains, whereas those with long cytoplasmic tail (2DL and 3DL) are inhibitory due to the presence of ITIMs (tyrosine-based inhibitory motifs), responsible for signal transduction in order to inhibit NK functions. Molecules with short cytoplasmic tail (2DS and 3DS) have an amino acid transmembrane region which allows the association with a particular protein (DAP12), which releases activating signals through ITAMs (tyrosine-based activation motifs) [15].

KIR receptors of NK cells may contribute to the occurrence of different immunological and clinical responses to the same disease in a specific population [16]. Several studies have described the participation of KIR (and their ligands) in infectious diseases, such as AIDS [17, 18], hepatitis C [19, 20], tuberculosis [21, 22], leprosy [23, 24] and malaria [25, 26, 27]. KIR are also involved in autoimmune and inflammatory diseases such as pemphigus foliaceus, psoriasis, scleroderma, rheumatoid vasculitis and Crohn’s disease [28, 29, 30, 31, 32], as well as in many types of cancer [33–36] and the survival of transplant patients [37]. The relationship between KIR and their HLA ligands in the immunopathogenesis of chronic chagasic disease remains unknown. Therefore, the aim of this study was to investigate the influence of the *KIR* genes and their HLA ligands in resistance or susceptibility to Chagas disease.

## Materials and Methods

This study was approved by the Human Research Ethics Committee of the Maringa State University (COPEP-UEM # 012/2010, CAAE 0296.0.093.000–09). All adults individuals who agreed to participate in this research were informed about the nature of the study and signed an informed consent form.

### Subjects

This case-control study enrolled 131 unrelated patients (CCD group) (59 men and 72 women, with a mean age of 60.4 ± 9.8 years) with serologically-diagnosed chronic Chagas disease, living in different municipalities in the north/northwest region of the State of Parana (located in the southern region of Brazil, between 22°29'30"-26°42'59"S and 48°02'24"-54°37'38"W) and treated at the University Hospital of Londrina and the Chagas Disease Laboratory of the State University of Maringa. A control group was formed of 165 healthy, unrelated individuals, who were spouses of the patients or blood donors of the Regional Blood Bank of Maringa (84 men and 81 women, with a mean age of 59.0 ± 11.4 years), living in the same geographical area as the patients and whose serological examination for antibodies against Chagas disease was negative. All individuals (patients and controls) that had participated in this study were monitored and evaluated for clinical symptoms and epidemiological data.

The inclusion criteria of the patient group were: positive laboratory diagnosis of Chagas disease and being in the chronic phase of the disease at the time of the study. The inclusion criteria for the control group were: negative laboratory diagnosis for Chagas disease and living in the same geographical region as the patients. The characteristics of the patients and controls are shown in Table 1.

**Table 1:** Characteristics of the chronic Chagas disease patients and control individuals.

	Control	CCD	NC	CCC
Characteristic				
	N = 165	N = 131	N = 87	N = 44
Age (years)				
Mean	59.0 ± 11.4	60.4 ± 9.8	59.0 ± 9.0	63.3 ± 10.5
Minimum	35	35	35	35
Maximum	98	87	87	85
Gender (n as %)				
Female	81 (49.1)	72 (55.0)	53 (60.9)	19 (43.2)
Male	84 (50.9)	59 (45.0)	34 (39.1)	25 (56.8)
Ethnic group* (n as %)				
White	114 (69.1)	96 (73.3)	62 (71.3)	34 (77.3)
Mulatto	36 (21.8)	23 (17.5)	17 (19.5)	6 (13.6)
Black	15 (9.1)	12 (9.2)	8 (9.2)	4 (9.1)
Cardiopathy (n as %)				
Yes		44 (36.6)		
No		87 (66.4)		

CCD: chronic Chagas disease patients; NC: without heart involvement patients, CCC: chronic chagasic cardiopathy patients.

*mixed population (white + mulatto + black) = 165 controls and 131 patients

Due to the great miscegenation in the Brazil population, and after considering the population composition of the state of Parana according to Probst *et al*. [38], patients and controls were classified as a mixed ethnicity population. The risk of population stratification bias, due to differences in ethnic background between patients and controls and variations of allele frequencies according to ethnic background, was minimized by matching patients with controls individuals of the same ethnic background. Mean age, gender rates and residence in the same geographical areas were carefully matching to select the groups.

### Serology for *T*. *cruzi*

Laboratory diagnosis of Chagas disease in patients and controls individuals was carried out by the Regional Blood Bank of Maringa with an ELISA (Enzyme-Linked ImmunoSorbent Assay) test of serum or plasma, using "Chagas III" reagents (GrupoBios, Santiago, Chile) following the manufacturer's instructions. The microplates were read using semi-automatic equipment (ASYS Expert Plus, Cambridge, UK). ELISA cut-off was defined by the formula: cut-off value = (average absorbance of the positive controls + average absorbance of the negative controls) x 0.35; and absorbance equal to or greater than the cut-off value was considered reactive. The indeterminate zone was defined by the absorbance values observed between the cut-off ± 10%. The samples were tested in duplicate and positive and negative controls were included. When the absorbance value was in the indeterminate zone, the ELISA test was repeated in duplicate and the indirect immunofluorescence (IFI) test was performed, according WHO recommendation. The Clinical Immunology Laboratory of the State University of Maringa performed an IFI test with the IMUNOCRUZI® antigen (Biolab, Rio de Janeiro, Brazil) and human anti-immunoglobulin G conjugated to fluorescein (Laborclin, Pinhais, Brazil). Samples were considered positive with titers ≥ 40.

### Heart examination

The patients with chronic Chagas disease (CCD group) were divided into two distinct groups according to the changes observed in the standard electrocardiography examination at rest. Of the all Chagas disease patients, 44 of them (36.6%), 25 men and 19 women, mean age of 63.3 ± 10.5 years, were considered to have chronic chagasic cardiopathy (CCC group) as they had cardiac signs characteristic of Chagas disease such as right bundle branch block, left anterior hemi-block, unspecific ventricular repolarisation disorders and ventricular and supraventricular premature beats. The remaining 87 (66.4%) patients with chronic Chagas disease, 34 men and 53 women, with a mean age of 59.0 ± 9.0 years, were considered as not having heart disease (NC group) (Table 1).

### KIR and HLA genotyping

DNA from all samples was extracted by the salting-out method adapted by Cardozo *et al*. [39]. The concentration and purity of DNA were verified using NanoDrop 2000 equipment (Thermo Scientific, Wilmington, USA). KIR and HLA-A, B and C were genotyped according manufacturer’s instructions by Polymerase Chain Reaction-Sequence Specific Oligonucleotide Probes protocols with Luminex technology (One Lambda Inc., Canoga Park, CA, USA). First, target DNA was PCR-amplified using group specific primers sets. Each PCR product was biotinylated, which allowed later detection using R-Phycoerythrin-conjugated Strepavidin (SAPE). Each PCR product was denatured and allowed to hybridise to complementary DNA probes conjugated to fluorescently coded microspheres. After washing the beads, bound amplified DNA from the test samples was tagged with SAPE. A flow analyser, the LABScan 100, identified the fluorescent intensity of PE (phycoerithrin) on each microsphere. The fluorescent intensity varied based on reaction outcome, and was expected to be 1000 or above for control positive probes. The data were interpreted using a computer program (HLA Fusion 2.0 Research, One Lambda).

Some HLA-KIR ligands specificities belonging to the group C1, C2 and Bw4 were considered according to Kulkarni *et al*. [40] and Thananchai *et al*. [41]. HLA molecules from the C1 group include the specificities from HLA-C*01, *03, *07, *08, *12, *14 *16 and are ligands of KIR2DL2, KIR2DL3 and KIR2DS2. HLA molecules from C2 group that include HLA-C*02, *04, *05, *06, *07,*15, *17, and *18 specificities interact with KIR2DL1 and KIR2DS1. HLA-Bw4 epitopes (HLA-A*23, *24, *32; HLA-B*13, *27, *44, *51, *52, *53, *57, *58) are recognized by KIR3DL1 and KIR3DS1. Specificities from HLA-A*03 and/or—A*11 are KIR3DL2 ligands.

Based on the content of the genes, two types of KIR genotypes have been described and are designated AA and BX (BB and AB). The main distinction between them is the number of genes encoding activating receptors. Individual genotypes were determined to be AA when the genes *KIR2DL1*, *KIR2DL3*, *KIR2DL4*, *KIR2DS4*, *KIR3DL1*, *KIR3DL2*, *KIR3DL3*, *KIR2DP1* and *KIR3DP1* were present. The presence of one or more of the following genes: *KIR2DL5*, *KIR2DS1*, *KIR2DS2*, *KIR2DS3*, *KIR2DS5* and *KIR3DS1* characterised the genotype BX (defined according http://www.allelefrequencies.net).

### Statistical analysis

*KIR*, *HLA* and KIR-HLA frequencies were obtained by direct counting. Comparisons of the frequencies of KIR ligands, *KIR* genes, KIR AA and BX genotypes and KIR with or without ligands between patients and controls were performed using the Chi-square test with Yates’ correction or Fisher's Exact Test. The associations of genetic trait between chronic Chagas disease and controls were measured by OR (Odds Ratio) and the 95% confidence interval (95% CI). Statistical analyses were performed using the Open Epi program: Open Source Epidemiologic Statistics for Public Health, version 2.3.1 (http://www.openepi.com/Menu/OE_Menu.htm). *P*-values ≤ 0.05 were considered statistically significant. A correction for multiple testing was done by multiplying the *P*-values by the number of the tests (Bonferroni correction). A Hardy-Weinberg equilibrium fit was performed by calculating expected genotype frequencies and comparing with the observed values using Arlequin software (version 3.1).

## Results

*HLA* and *KIR* genotypes frequency distribution in the studied populations was in Hardy-Weinberg equilibrium. The frequencies of *KIR* genes in the control group were similar to those found by Rudnick *et al*. [42] in individuals of the north/northwest region of the state of Paraná.

Distribution of *KIR* gene frequencies among controls, chronic Chagas disease patients (CCD), patients without heart involvement (NC), and chronic chagasic cardiopathy patients (CCC) is shown in Table 2. CCC presented a lower frequency of *KIR2DL2* when compared to the control group (31.8% vs. 53.3%; *P* = 0.017; OR = 0.41; 95% CI = 0.20–0.83); however, significance was lost after Bonferroni correction (*Pc* = 0.27). No significant differences were found in the distribution of other *KIR* genes between all of the analysed groups. According to expected the *KIR* framework genes, *KIR2DL4*, *KIR3DL2*, *KIR3DL3* and *KIR3DP1* were present in all samples, which were important internal controls.

**Table 2:** Distribution of *KIR* genes in healthy controls, chronic Chagas disease patients and in groups of patients with and without heart involvement.

	Control	CCD	NC	CCC
KIR genes	N = 165	N = 131	N = 87	N = 44
	n (%)	n (%)	n (%)	n (%)
*KIR2DL1*	162 (98.2)	131 (100)	87 (100)	44 (100)
*KIR2DL2*	88 (53.3) ^**a**^	49 (37.4)	35 (40.2)	14 (31.8) ^**a**^
*KIR2DL3*	146 (88.5)	119 (90.8)	79 (90.8)	40 (90.9)
*KIR2DL4*	165 (100)	131 (100)	87 (100)	44 (100)
*KIR2DL5*	102 (61.8)	73 (55.7)	53 (60.9)	20 (45.5)
*KIR2DP1*	162 (98.2)	130 (99.2)	87 (100)	43 (97.7)
*KIR2DS1*	67 (40.6)	57 (42.7)	43 (49.4)	14 (31.8)
*KIR2DS2*	92 (55.8)	62 (48.1)	40 (46.0)	22 (50.0)
*KIR2DS3*	59 (35.8)	29 (22.1)	17 (19.5)	12 (27.3)
*KIR2DS4*	155 (93.9)	124 (94.7)	81 (93.1)	43 (97.7)
*KIR2DS5*	69 (41.8)	59 (41.8)	44 (50.6)	15 (34.1)
*KIR3DL1*	158 (95.8)	127 (96.9)	84 (96.6)	43 (97.7)
*KIR3DL2*	165 (100)	131 (100)	87 (100)	44 (100)
*KIR3DL3*	165 (100)	131 (100)	87 (100)	44 (100)
*KIR3DP1*	165 (100)	131 (100)	87 (100)	44 (100)
*KIR3DS1*	63 (38.2)	54 (41.2)	41 (47.1)	13 (29.5)

CCD: chronic Chagas disease patients, NC: without heart involvement patients, CCC: chronic chagasic cardiopathy patients.

^**a**^ *P* = 0.017; *Pc* = 0.27; OR = 0.41; 95% CI = 0.20–0.83 (CCC vs Controls)

The frequencies of the HLA class I ligands of the KIR (A3 and/or A11, Bw4, C1 and C2, in homozygosity and heterozygosity) were analysed and were similar between groups. An exception was found for the specificities of the HLA-A*03 and/or—A*11, ligands of KIR3DL2, which were lower in chronic Chagas disease patients (CCD) (19.1% vs. 30.3%; *P* = 0.036; *Pc* = 0.144; OR = 0.54; 95% CI = 0.31–0.94), but the significance was lost after Bonferroni correction.

The distribution of the frequencies of the KIR and their HLA ligands (KIR2DL2-C1; KIR3DL3-C1; KIR2DS2-C1; KIR2DL1-C2; KIR2DS1-C2; KIR3DL1-Bw4, KIR3DS1-Bw4) are listed in Table 3. The KIR2DL2-C1 (16.8% vs. 32.1%; *P* = 0.036; OR = 0.43; 95% CI = 0.24–0.75), the KIR3DL2-A3/11 pair (19.1% vs. 30.3%; *P* = 0.037; OR = 0.54; 95% CI = 0.31–0.94) and KIR2DL2 in the presence of the ligands in the homozygous state (KIR2DL2-C1/C1) (4.5% vs. 19.5% *P* = 0.031; OR = 0.23; 95% CI = 0.04–0.89) had lower frequency in the patients with chronic Chagas disease, but the significance was lost after Bonferroni correction.

**Table 3:** Distribution of KIR and their respective HLA ligands in Chagas disease patients and controls.

	Control	CCD	NC	CCC
KIR—HLA ligands	N = 165	N = 131	N = 87	N = 44
	n (%)	n (%)	n (%)	n (%)
2DL1-C2	80 (48.4)	57 (43.5)	35 (40.2)	22 (50.0)
2DL2-C1	53 (32.1) ^**a**^	22 (16.8) ^**a**^	14 (16.1)	9 (22.5)
2DL3-C1	75 (45.5)	54 (41.2)	33 (37.9)	21 (47.7)
3DL2-A3/A11	50 (30.3) ^**b**^	25 (19.1) ^**b**^	18 (20.7)	7 (15.9)
3DL1-Bw4	113 (68.5)	94 (71.8)	60 (69.0)	34 (77.3)
2DS1-C2	37 (22.4)	22 (16.8)	16 (18.4)	6 (13.6)
2DS2-C1	53 (32.1)	27 (20.6)	15 (17.2)	12 (27.3)
3DS1-Bw4	47 (28.5)	40 (30.5)	29 (33.3)	11 (25.0)
2DL1-C2C2	40 (24.2)	25 (19.1)	19 (21.8)	6 (13.6)
2DL2-C1C1	19 (11.5)	19 (14.5)	17 (19.5) ^**c**^	2 (4.5) ^**c**^
2DL3-C1C1	37 (22.4)	43 (32.8)	30 (34.5)	13 (29.5)
2DS1-C2C2	13 (7.9)	14 (10.7)	13 (14.9)	1 (2.3)
2DS2-C1C1	20 (12.1)	26 (19.8)	20 (23.0)	6 (13.6)
2DL2/2DL2-C1C2	9 (5.5)	2 (1.5)	1 (1.1)	1 (2.3)
2DL2/2DL3-C1C2	44 (26.7)	20 (15.3)	12 (13.8)	8 (18.2)
2DL3/2DL3-C1C2	31 (18.8)	34 (26.0)	21 (24.1)	13 (29.5)
2DL2/2DL2-C1C1	3 (1.8)	6 (4.6)	5 (5.7)	1 (2.3)
2DL2/2DL3-C1C1	16 (9.7)	13 (9.9)	12 (13.8)	1 (2.3)
2DL3/2DL3-C1C1	21 (12.7)	30 (22.6)	18 (20.7)	12 (27.3)

CCD: chronic Chagas disease patients; NC: without heart involvement patients, CCC: chronic chagasic cardiopathy patients.

^**a**^ *P* = 0.036; *Pc* = 0.108; OR = 0.43; 95% CI = 0.24–0.75 (CCD vs Controls)

^**b**^ *P* = 0.037; *Pc* = 0.10; OR = 0.54; 95% CI = 0.31–0.94 (CCD vs Controls)

^**c**^ *P* = 0.031; *Pc* = 0.093; OR = 0.23; 95% CI = 0.04–0.89 (CCC vs NC)

Bw4 = HLA-A*23,*24,*32; HLA-B,*13,*27,*44,*51,*52,*53,*57,*58

Group C1 = HLA-C*01,*03,*07,*08,*12,*14,*16

Group C2 = HLA-C*02,*04,*05,*06,*07,*15,*17,*18

The correlation between the distribution of activating and inhibitory KIR and their respective HLA ligands is shown in Table 4. An increased risk or susceptibility of developing chronic Chagas disease (12.2% vs. 4.2%; *P* = 0.020; *Pc* = 0.040; OR = 2.14; 95% CI = 1.25–7.88) and chronic chagasic cardiopathy (22.7% vs. 4.2%; *P* = 0.0002; *Pc* = 0.0004; OR = 6.64; 95% CI = 2.30–18.60) was observed for the KIR2DS2^+^/2DL2^-^/C1^+^ combination (KIR2DS2 and the C1 ligand in the absence of KIR2DL2). This correlation was also observed when CCC was compared to the NC (22.7% vs. 6.9%; *P* = 0.010; *Pc* = 0.020; OR = 3.97; 95% CI = 1.34–11.79). Susceptibility was also observed when KIR2DL3 was present, KIR2DS2^+^/2DL2^-^/KIR2DL3^+^/C1^+^ combination, for CCD (10.7% vs. 4.2%; *P* = 0.050; *Pc* = 0.10; OR = 1.06; 95% CI = 1.1–6.9) and when CCC was compared to NC (18.2% vs. 5.7%; *P* = 0.041; *Pc* = 0.08; OR = 3.64; 95% CI = 1.12–11.91), although it was lost after Bonferroni correction. However, the susceptibility of developing disease remained for CCC (18.2% vs. 4.2%; *P* = 0.004; *Pc* = 0.004; OR = 5.02; 95% CI = 1.71–14.73) when compared to controls.

**Table 4:** Distribution of activating KIR plus inhibitory KIR and their respective ligands in chronic Chagas disease and controls.

		Control	CCD	NC	CCC
KIR-HLA		N = 165	N = 131	N = 87	N = 44
Ligand		n (%)	n (%)	n (%)	n (%)
KIR-C1					
	2DS2^+^/2DL2^-^/C1^+^	7 (4.2) ^**a**^ ^**,**^ ^**b**^	16 (12.2) ^**a**^	6 (6.9) ^**c**^	10 (22.7) ^**b**^ ^**,**^ ^**c**^
	2DS2^-^/2DL2^+^/C1^+^	6 (3.6)	4 (3.1)	1 (1.1)	3 (6.8)
	2DS2^+^/2DL3^-^/C1^+^	12 (7.3)	9 (6.9)	6 (6.9)	3 (6.8)
	2DS2^-^/2DL3^+^/C1^+^	51 (30.0)	53 (40.5)	34 (39.1)	19 (43.2)
	2DS2^+^/2DL2^-^/2DL3^-^/C1^+^	0 (0.0)	2 (1.5)	0 (0.0)	2 (4.5)
	2DS2^-^/2DL2^-^/2DL3^+^/C1^+^	45 (27.3)	50 (38.2)	33 (37.9)	17 (48.6)
	2DS2^+^/2DL2^-^/2DL3^+^/C1^+^	7 (4.2) ^**d**^ ^**,**^ ^**f**^	14 (10.7) ^**d**^	5 (5.7) ^**e**^	8 (18.2) ^**e**^ ^**,**^ ^**f**^
	2DS2^-^/2DL2^+^/2DL3^+^/C1^+^	5 (3.)	3 (2.3)	1(1.1)	2 (4.5)
	2DS2^+^/2DL2^+^/2DL3^-^/C1^+^	12 (7.3)	7 (5.3)	6 (6.9)	1 (2.3)
	2DS2^+^/2DL2^+^/2DL3^+^/C1^+^	54 (32.7)	30 (22.9)	23 (26.4)	7 (15.9)
KIR- C2					
	2DS1^+^/2DL1^-^/C2^+^	3 (1.8)	0 (0.0)	0 (0.0)	0 (0.0)
	2DS1^-^/2DL1^+^/C2^+^	70 (42.4)	43 (32.8)	26 (29.9)	21 (47.7)
	2DS1^+^/2DL1^+^/C2^+^	47 (28.5)	36 (27.5)	29 (33.3)	7 (15.9)
KIR- Bw4					
	3DS1^+^/3DL1^+^/Bw4^+^	41 (24.8)	35 (26.7)	25 (28.7)	10 (22.7)
	3DS1^+^/3DL1^-^/Bw4^+^	7 (4.2)	3 (2.3)	2 (2.3)	1 (2.3)
	3DS1^-^/3DL1^+^/Bw4^+^	72 (43.6)	60 (46.3)	36 (41.4)	24 (54.5)
KIR-HLA- A3/11					
	3DL2^+^/A3^+^/A11^+^	50 (30.3) ^**g**^	25 (19.1) ^**g**^	18 (20.7)	7 (15.9)
	3DL2^+^/A3^-^/A11^+^	17 (10.6)	6 (4.6)	5 (5.8)	1 (2.3)
	3DL2^+^/A3^+^/A11^-^	33 (20.0)	19 (14.5)	13 (14.9)	6 (13.6)
	3DL2^+^/A3^-^/A11^-^	118 (71.5)	106 (80.9)	69 (79.3)	37 (84.1)

CCD: chronic Chagas disease patients; NC: without heart involvement patients, CCC: chronic chagasic cardiopathy patients.

^**a**^ *P* = 0.020; *Pc* = 0.040; OR = 2.14; 95% CI = 1.25–7.88 (CCD vs Controls)

^**b**^ *P* = 0.0002; *Pc* = 0.0004; OR = 6.64; 95% CI = 2.30–18.60 (CCC vs Controls)

^**c**^ *P* = 0.010; *Pc* = 0.020; OR = 3.97; 95% CI = 1.34–11.79 (CCC vs NC)

^**d**^ *P* = 0.050; *Pc* = 0.100; OR = 1.06; 95% CI = 1.1–6.9 (CCD vs Controls)

^**e**^ *P* = 0.040; *Pc* = 0.080; OR = 3.64; 95% CI = 1.12–11.91 (CCC vs NC)

^**f**^ *P* = 0.004; *Pc* = 0.008; OR = 5.02; 95% CI = 1.71–14.73 (CCC vs Controls)

^**g**^ *P* = 0.036; *Pc* = 0.144; OR = 0.54; 95% CI = 0.31–0.94 (CCD vs Controls)

No significant difference was observed in the AA and BX genotype frequencies between all groups. Also, there was no significant difference in the frequencies of AA genotypes when the complete forms of the *KIR2DS4* gene or its variants (deleted form) were present.

## Discussion

To the best of our knowledge, this is the first study of KIR and the HLA ligands in the immunopathology of chronic Chagas disease. The identification of the role of KIR and KIR-HLA ligand pairs in the Chagas disease development could improve our understanding of the role of NK cells in the immunopathogenesis of this disease.

The current study shows that the distribution of the *KIR* genes highlights differences in the frequency of *KIR2DL2* when chronic chagasic cardiopathy patients (CCC) were compared to controls, although the significance was lost after correction for multiple testing. *KIR2DL2* and *KIR2DL3* segregate as alleles of a single locus, and are both common and recognise C1 [43]. Although KIR2DL2 and KIR2DL3 exhibit quantitative differences in specificity and avidity for the HLA-C1 ligand, they qualitatively differ in their genetics, functional effect, and clinical influence [44, 45]. In the current study, *KIR2DL3* was not significantly different in any of the comparisons, revealing a possible distinction between these receptors in NK-cell regulation in the cardiac form of Chagas disease.

KIR were analysed in the presence of their respective HLA ligands. HLA class I genes are located on chromosome 6 and *KIR* genes are on chromosome 19, which allows independent inheritance and expression of both. The independent segregation of these genes, coupled with the high specificity of KIR for certain HLA allotypes, allows the expression of KIR molecules for which the HLA ligand is not present, or *vice versa*; this results in a lack of functionality of NK cells due to a lack of signalling [40]. Moreover, depending on the combination of KIR and HLA ligands, NK cells may exhibit different degrees of response due to an excess of inhibition or activation, a balance between inhibition and activation, or even an undetermined behaviour [46, 47].

In this study, the pair KIR3DL2-HLA-A3/A11 was less frequent in chronic Chagas disease: *KIR3DL2* is a framework gene that is present in almost all genotypes, meaning that the difference in the frequency of this inhibitory KIR-HLA pair may be related to differences in the frequencies of HLA-A3/A11 ligands and not reflecting participation in the pathogenesis of Chagas disease.

However, the results indicate that the interaction of KIR2DL2 and KIR2DS2 with HLA-C1 may have an important role of NK cells in the development of Chagas disease. KIR2DS2 and KIR2DL2 share the same ligand most likely because of the homology of the same extracellular portion, although there is a difference in the function of activating or inhibiting NK cells, respectively. In this study, KIR2DL2 in the presence of the HLA-C1 ligand was less frequent in chronic Chagas disease patients than in controls and those patients with chronic chagasic cardiopathy also had a lower frequency of KIR2DL2 with the homozygous C1 (KIR2DL2-C1/C1) compared to the group without heart failure, although the significance was lost after correction for multiple testing. No association of KIR2DS2-C1 was found between Chagas disease and controls.

To evaluate the C1-reactive KIR2D NK cells, the combination of one pair (inhibitory) in the absence of the other pair (activating) was analysed (the KIR2DL3 was included as well). The results are shown in Table 4. It was possible to observe that KIR2DS2-C1 in the absence of KIR2DL2 (KIR2DS2^+^/2DL2^-^/C1^+^ combination) was more frequent in Chagas patients and, in particular, in those who manifested chronic chagasic cardiopathy when compared to the control group, and when chronic chagasic cardiopathy patients were compared to the without heart involvement patients. In addition, KIR2DS2^+^ in the absence of KIR2DL2 and the presence of KIR2DL3 (KIR2DS2^+^/2DL2^-^/KIR2DL3^+^/C1^+^) was also significantly more common in the CCC group compared to the NC group.

The data obtained in this study indicated a possible susceptibility related to the activating KIR2DS2 and its C1 ligand in the absence of KIR2DL2, for the development of Chagas disease and chronic chagasic cardiopathy. The KIR2DL2-C1 pair has a strong inhibitory effect on NK cells and the KIR2DL3-HLA-C1 pair caused weaker inhibitory signals [48]. Although the same ligand can have affinities for both activating (-DS) and inhibitory (-DL) receptors of NK cells for some KIR, the affinity for inhibitory KIR is higher than for its homologous activating KIR [46]. David *et al*. [49] showed that when KIR2DL2 and KIR2DS2 were co-expressed, NK cell inhibition overrode NK cell activation. Thus, if inhibitory KIR are absent from the surfaces of NK cells, group C HLA ligands will bind to activating receptors, inducing the effector function of the NK cells and consequently greater inflammation and tissue injury. This predisposes to chronic Chagas disease and the manifestations of chronic chagasic cardiopathy. Although the KIR2DS2^+^/2DL2^-^/KIR2DL3^+^/C1^+^ combination presents one activating and one inhibitory KIR, the activating signals generated by KIR2DS2^+^/C1^+^ appeared to be stronger than the inhibitory signals generated by KIR2DL3^+^/C1^+^. In other studies, the KIR2DL2-C1 pair was associated with protection against chronic myeloid leukaemia [50] systemic sclerosis [51] and kidney cancer patients [52] and KIR2DS2 (KIR2DS2^+^/2DL2^-^/C1^+^) was more common for other autoimmune diseases [30, 53].

The *KIR2DL2* and *KIR2DS2* genes are in strong linkage disequilibrium, as observed among patients (Δ' = 0.99; *P*-value = 0.004) and controls (Δ' = 1.0; *P*-value = 0.0001). However, in this study, there were 8 controls (4.8%) and 4 patients (3.1%) where only *KIR2DL2* was present, and 10 controls (6.1%) and 18 patients (13.7%) with *KIR2DS2* (activating) but without *KIR2DL2* (inhibitory). Within the sub-groups, three patients with cardiopathy (6.8%) and one without heart disease (1.2%) had only *KIR2DL2*, whilst *KIR2DS2* alone was present in 11 patients with cardiopathy (25.0%) and 7 patients without heart disease (8.1%).

The immune response during infection by *T*. *cruzi* determines the development of the different clinical manifestations of Chagas disease: it is associated with both the pathogenesis and protective effects that control tissue damage [12]. Several studies have shown that there is an increase in the frequency of circulating NK cells in chronic chagasic cardiopathy patients, and uncontrolled activation of NK cells and pro-inflammatory monocytes can also lead to tissue damage, which, in turn, leads to the development of serious chronic illness [11, 12, 13]. For other hand, Chagas cardiopathy could be linked to autoimmunity [9, 54, 55]. In this study, we investigated KIR-HLA ligand as a risk factor in Chagas disease and these results could corroborate to the NK immunopathogenic mechanism in the Chagas disease understanding.

In this KIR-HLA ligands study, a possible risk factor for the development of Chagas disease and chronic chagasic cardiopathy related to the activating KIR2DS2 and its C1 ligand in the absence of KIR2DL2 was found. To better understanding the role of NK cells and the expression or KIR in the immunopathogenesis of Chagas disease, others studies would be done like histological analysis in the CCC and NK cytotoxicity assays.

### Conclusion

The combination of KIR2DS2 activating receptor with C1 ligand, in the absence of KIR2DL2, may be related to a risk factor in the chronic Chagas disease and chronic chagasic cardiopathy.
